# The incidence of detectable bacterial contaminated primary joint arthroplasties and periprosthetic joint infection: a systematic review and meta-analysis

**DOI:** 10.1530/EOR-2025-0074

**Published:** 2026-06-01

**Authors:** Maarten M Bruin, Rob G H H Nelissen, Jan W Schoones, Mark G J de Boer, Ruud L M Deijkers, Bart G C W Pijls

**Affiliations:** ^1^ Department of Orthopedics, Leiden University Medical Center, Leiden, The Netherlands; ^2^ Directorate of Research Policy, Leiden University Medical Center, Leiden, The Netherlands; ^3^ Department of Infectious Diseases, Leiden University Medical Center, Leiden, The Netherlands; ^4^ Department of Orthopedic Surgery, HagaZiekenhuis, The Hague, The Netherlands

**Keywords:** PJI, prosthetic joint infection, contamination, contaminated joint arthroplasty, revision

## Abstract

**Purpose:**

- The association between positive intraoperative cultures during primary joint arthroplasty and periprosthetic joint infections (PJIs) remains controversial. This systematic review and meta-analysis examines the association between intraoperative contamination and complications such as PJIs and revision rates.

**Methods:**

- We searched PubMed, Web of Science, Cochrane, Emcare (OVID), Embase (OVID), MEDLINE, Academic Search Premier and the references of included studies evaluating the outcome of contaminated and non-contaminated primary joint arthroplasties. This systematic review and meta-analysis was performed according to the PRISMA statement, and the research protocol was uploaded *a priori* at Open Science Framework. Two researchers independently reviewed all the titles, abstracts and full texts, and extracted the available data. Study eligibility criteria were primary joint arthroplasty with cultures taken during initial procedure and documentation of PJIs and revision rates.

**Results:**

- The search yielded 26 studies after inclusion and exclusion criteria, involving 4,556 primary joint arthroplasties. The overall incidence of bacterial contamination was 24.7% (95% CI: 19–30%). The incidence of PJIs was 2.0% (95% CI: 1.1–2.8%) in the contaminated group versus 1.0% (95% CI: 0.6–1.4%) in the non-contaminated group, with a pooled adjusted risk ratio (RR) of 2.0 (95% CI: 1.3–3.1). The pooled analysis revealed an increased risk of contaminated joint arthroplasties for revision for any reason, with a pooled RR of 1.86 (95% CI: 1.14–3.03).

**Conclusion:**

- This systematic review and meta-analysis suggests intraoperative bacterial contamination may increase the risk of PJIs and the risk of revision in primary hip, knee and shoulder arthroplasty. However, over more than 9 out of 10 patients with an intraoperative contaminated joint arthroplasty did not develop a PJI.

## Introduction

Periprosthetic joint infections (PJIs) are a major complication in joint arthroplasty, a procedure frequently performed and increasingly common in today’s aging society (1). In 2010 alone, surgeons in the United States performed a total of 719,000 total knee arthroplasties (TKAs) and 572,000 total hip arthroplasties (THAs). The overall prosthetic joint infection rates settled around 2% for the United States (2001–2009), with evidence of a potential increase since the study of Tande and Patel (1). Therefore, prevention of PJIs is vital in joint arthroplasty.

In PJIs, usually a microorganism (i.e. bacteria and fungus) is accumulated around the prosthesis, forming a biofilm within the first days after contamination, eventually manifesting in a PJI. PJIs or surgical site infections (SSIs) occur when the microbial load at the affected tissue or prosthesis site surpasses the ability of the host immune response to eliminate the pathogens (2) of a broad spectrum of variety in every patient. Intraoperative contamination remains the most recognized pathway for PJIs (3). Even chronic PJIs can arise from intraoperative contamination by less virulent bacteria (low grade) (4), thus over time prompting surgeons to create precautions, all aimed at reducing the risk of PJIs even more, such as laminal flow, antibiotic prophylaxis, skin disinfectants, wound protectors, skin glue, sterile dressings/gown and sterilized instruments and prostheses (1).

Multiple risk factors for PJIs have been described; some risk factors (i.e. diabetes mellitus, obesity and smoking) support the idea of compromised soft tissues and immune host response, leading to a greater risk of infection. Three main mechanisms of contamination causing a PJI/SSI are intraoperative contamination, hematogenous spread of pathogens (i.e. bacteremia) and contamination from adjacent infected tissues (5). Furthermore, translocation of bacteria to an artificial joint or surgical sites could be done by neutrophils and macrophages, which have encapsulated bacteria, such as a Trojan horse (2).

The current assumption that most SSIs after elective surgery under standard methods of antisepsis and antibiotic prophylaxis are due to intraoperative contamination remains unproven (2). Therefore, the aim of this study was to evaluate whether an association exists between (detectable) intraoperative contamination and PJIs in a systematic review and meta-analysis. Secondarily, we evaluated the presence of the same microorganism during primary joint arthroplasty and clinically evident PJIs.

## Materials and methods

This systematic review and meta-analysis was performed according to the PRISMA statement, and the research protocol was uploaded *a priori* at Open Science Framework (https://doi.org/10.17605/OSF.IO/87S64). The population consisted of patients undergoing primary joint arthroplasty for primary or secondary osteoarthritis (studies focusing on tumors or acute trauma were excluded). The exposed group consisted of patients with at least one positive intraoperative culture during primary surgery. The control group consisted of patients with negative intraoperative cultures during primary surgery. The primary outcome was the incidence of PJIs. In addition, we evaluated whether there was a match for microorganisms found during primary arthroplasty and clinically evident PJIs.

### Data sources and searches

The search strategy was designed in cooperation with an information specialist (JS) and was composed of primary joint arthroplasty, with cultures taken during the primary procedure to determine contamination, PJI rates and revision rates depending on contamination status. The full search strategy is included in the Supplementary file (see section on Supplementary materials given at the end of the article). We searched the following databases: PubMed, Web of Science, Cochrane, Emcare (OVID), Embase (OVID), MEDLINE and Academic Search Premier. In addition, the references of the included studies were screened for possible inclusions. The search results were imported into endnote, and the duplicates were identified and removed.

### Study selection

The literature was screened independently by two reviewers (MB and BP) on title and abstract to identify studies that potentially meet the inclusion criteria. Both the reviewers documented their findings in a form that was designed before the study started. The results were compared, and any disagreements were resolved by consensus or by consulting a referee. The full-text papers of eligible studies were evaluated independently by two reviewers and recorded. Subsequently, disagreements were solved by consensus or by consulting a referee. The following inclusion criteria were used to identify eligible studies: i) primary joint arthroplasty, ii) primary or secondary osteoarthritis as a reason for joint arthroplasty, iii) intraoperative cultures taken during primary joint replacement surgery, iv) PJIs/SSIs evaluated and v) languages spoken by the review team (English and Dutch). The exclusion criteria are as follows: i) prior history of infection of the joint, ii) revision arthroplasty, iii) postoperative (prolonged) treatment with antibiotics (this could influence the outcome of developing PJIs) and iv) no intraoperative cultures or infection rates evaluated.

### Data extraction and quality assessment

Two reviewers (MB and BP) extracted the data from the included studies regarding the number of patients/arthroplasties, incidence of PJIs, type of arthroplasty, reason for arthroplasty, patient demographics (e.g. age, sex, body mass index (BMI) and diabetes), antibiotic prophylaxis, location of cultures, revision rate, follow-up and study characteristics. We classified the culture samples/tissues into different categories: skin, soft tissues, periarticular and articular. Disagreements between the two databases were resolved by consensus or by consulting a referee (RN).

Risk of bias and quality of the included studies were individually assessed by the two reviewers using the Newcastle–Ottawa scale (NOS) (6). This scale is developed to assess the quality of non-randomized studies (including case–control and cohort studies). Randomized controlled trials were assessed with the NOS since our research question is observational. Also there was no randomization (in the randomized controlled trials (RCTs)) between contamination or no contamination (our research question). The randomization was for other factors such as skin preparation. We will not evaluate those groups (i.e. skin preparation) separately, but the RCT as a cohort. Disagreements were resolved by either consensus between the two reviewers or by consulting a referee.

### Statistical analysis

The data from the included studies were pooled in a meta-analysis to estimate the overall risk by the use of a random-effects model according to DerSimonian and Laird (7). Risk ratios were calculated for patients with positive versus negative intraoperative cultures on the following outcomes: PJIs, revision surgery and wound complications. Forest plots and between-study variance analysis were used for inspection to determine the amount of statistical heterogeneity (8). A funnel plot was constructed to assess publication bias in the studies reporting the primary outcome. In the presence of asymmetry in the funnel plot or potential publication bias, a trim-and-fill method was used to evaluate the size and effect (8, 9). All analyses were performed using the metafor package in R statistics (9).

In case of heterogeneity (*I*^2^ more than 40%) and when the number of studies allowed it, subgroup analyses or meta-regression will be performed on the following possible effect modifiers: type of arthroplasty, antibiotic prophylaxis and demographic factors (8).

## Results

### Study selection

**Figure 1:**
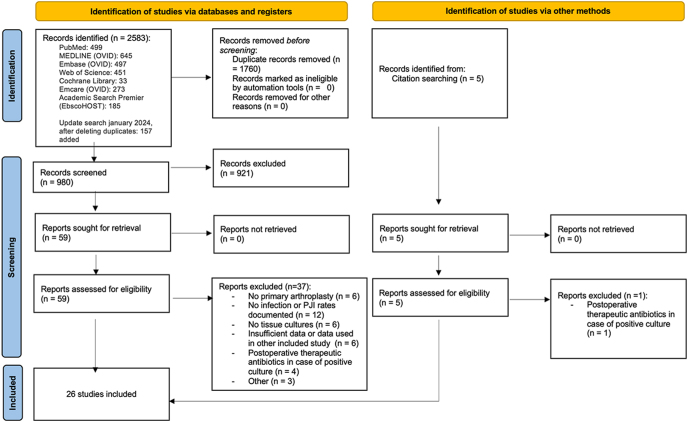
Flow chart of the study selection.

The flow chart of study inclusion is shown in Fig. 1. The initial search yielded 2,583 records, which increased to 2,740 records after an update in January 2024. After removing duplicates, 980 records were left for screening of the titles and abstracts. After screening, 59 studies remained for full-text screening. Thirty-seven studies were excluded as they did not meet the inclusion criteria; see Fig. 1. A search through the reference list of the included studies yielded five extra studies, with four studies meeting the inclusion and exclusion criteria. Conclusively, a total of 26 studies published between 2000 and 2024 met the inclusion and exclusion criteria and were included in our meta-analysis.

### Characteristics of included studies

The details of the included studies are shown in Table 1. Of the 26 included studies, four were RCTs and 22 were observational studies. The mean age ranged from 50 to 81 years. The follow-up ranged from one month to 156 months. In 14 studies, the patients had a follow-up of at least 12 months. Overall, 4,556 arthroplasties were analyzed in the meta-analysis; the number of arthroplasties per study ranged from 40 to 714. Seven studies were on total shoulder arthroplasty (*n* = 663 TSAs), 14 studies on THA or hemi-arthroplasty (*n* = 2,276 THAs and *n* = 143 hemi-arthroplasties), and nine studies on TKA (*n* = 1,474 TKAs). The contaminated group was on average 1.3 years younger and had fewer females than the non-contaminated group; other baseline characteristics were comparable (Table 2).

**Table 1:** Details of included studies.

Study	Study design	All					Contamination					No contamination				
		Number of			Age*	BMI*	Number of			Age*	BMI*	Number of			Age*	BMI*
		Shoulders	Hips	Knees			PTS (F)	WPs	PJIs			PTS (F)	WPs	PJIs		
Byrne (5)	PS	0	55	23			18	0	0			62	1	0		
Chalmers *et al.* (21)	PS	61	0	0	69.0 (8.0)	31.3 (8.2)	25 (11)		1			36 (21)		0		
Chen *et al.* (22)	PS	0	0	125	65.7 (9.9)	31.2 (5.1)	10		0			115		0		
Droll *et al.* (23)	RCT	0	105	0	65.8 (11.0)	30.0 (5.6)	34		0			71		0		
Ferro *et al.* (24)	RS	0	426	0	50.2 (13.4)		54 (20)		5			372 (176)		11		
Font-Vizcarra *et al.* (25)	PS	0	402	0	65.1 (13.9)	28.0 (4.3)	41 (17)		2	62.5 (14.9)	29.9 (4.4)	361 (186)		13	65.4 (13.7)	27.9 (4.4)
Font-Vizcarra *et al.* (26)	PS	0	93	0	81.0		29 (21)		2	83.6 (6.3)		64 (54)		2	79.8 (8.3)	
Gates *et al.* (27)	RS	83	0	0	64.2 (10.9)	29.8 (5.1)	37 (10)		0	63.8 (12.8)	30.1 (5.2)	46 (19)		0	64.5 (9.2)	29.6 (5.0)
Gates *et al.* (27)	RCT	60	0	0	72.2	29.0	11 (1)		0	67.5 (8.4)	28.7 (3.5)	49 (30)		0	73.3 (8.3)	29.0 (6.2)
Haenle *et al.* (29)	RS	0	0	206			89		4			117		2		
Hanada *et al.* (30)	PS	0	107	74	66.2	25.3 (4.0)	11		1			170		0		
James & Gower (31)	RS	0	60	0	68.0		24 (11)	2	1	68.00		26 (14)	4	0	68.0	
Jonsson *et al.* (32)	PS	0	49	41	69.0	27.0	41 (21)	1	0	69.0 (10.8)	27.0 (5.8)	49 (29)	0	1	69.0 (9.3)	27.0 (4.3)
Justesen *et al.* (33)	PS	0	0	714	68.0 (9.0)	29.0 (5.0)	84 (37)		2	67.0 (9.0)	29.0 (5.0)	630 (352)		10	68.0 (9.0)	29.0 (5.0)
Kim *et al.* (34)	PRCT	64	0	0	69.1 (7.9)	32.5 (8.0)	10		0			54		1		
Knobben *et al.* (35)	PS	0	100	0	61.3 (12.8)	27.0 (3.7)	36	20	6			64	8	1		
Pattyn *et al.* (36)	PS	0	100	0	63.1 (14.3)		16		0			84		0		
Phuong *et al.* (37)	RS	0	273	0	68.8		16		0			258		1		
Rattanaprichavej *et al*. (38)	RS	0	0	183	63.9 (8.6)	27.2 (4.0)	8 (6)		0	65.1 (10.0)	27.3 (2.9)	175 (142)		4	63.8 (8.6)	27.2 (4.1)
Rivera *et al.* (39)	PS	0	64	68	71.0 (8.6)	29.5	57 (26)		0	71.0 (9.6)	29.9 (5.1)	75 (49)		2	72.0 (7.9)	29.1 (4.9)
Sommerville *et al.* (40)	PS	0	170	0	72.5 (15.0)		40 (18)		1	70.0 (10.0)		130 (72)			73.0 (12.0)	
Symonds *et al.* (41)	RCT	99	0	0	68.7 (6.8)	30.8 (5.8)	24		0			75				
Ibrahim *et al.* (42)	RS	0	415	0	77.0		207 (53)		2	76.0 (11.5)		208 (51)			78.0 (8.5)	
Torchia *et al.* (43)	PS	0	0	40	65.9 (9.4)	31.8 (5.9)	12 (7)		0	62.4 (7.9)	33.0 (7.4)	28 (14)			67.3 (9.7)	31.3 (5.3)
Torrens *et al.* (44)	RS	162	0	0	74.0 (6.0)	28.6 (6.6)	25 (12)		2	71.7 (7.1)	27.5 (4.6)	137 (124)			75.4 (6.2)	28.7 (4.4)
Zmistowski *et al.* (45)	RS	134	0	0	67.6 (13.9)		42 (11)		0	68.8 (1.8)		92 (55)	1		69.5 (1.3)	

* Values are mean (SD).

PJIs, prosthetic joint infections; BMI, body mass index; PS, prospective study; RS, retrospective study; RCT, randomized controlled trial; PRCT, prospective randomized controlled trial; WPs, wound problems; PTS (F), patients (female).

**Table 2:** Baseline differences between contaminated and non-contaminated patients.

	Studies (*k*)	Patients (*n*)	Difference	95% CI
Age (years)	13	2,738	−1.3	−2.5 to −0.1
Female (%)				
Total	16	3,225	−15	−22 to −8.2
Only hips and/or knees	11	2,725	−8.0	−13 to −2.6
BMI	9	1,866	0.37	−0.35 to 1.1
DOS (minutes)	5	1,521	2.8	−1.3 to 6.9
Smoker (%)	3	549	6.4	−7.7 to 2
DM (%)	5	1,064	−3.3	−7.3 to 0.7

DOS, duration of surgery.

**Table 3:** Details of perioperative cultures.

Study	Category	Location cultures
Byrne *et al.* (5)	Skin, periarticular, articular	Swab incision site, swab femoral neck at 30 min intervals, swab from fascia before closure, swab suture line prior to closure
Chalmers *et al.* (21)	Skin, articular	Swab underneath adherent dressing from skin, culture from incision edge along the dermis, culture from the humeral articular
Chen *et al.* (22)	Periarticular	Three culture samples from the infrapatellar (Hoffa’s) fat pad
Droll *et al.* (23)	Skin	Swab samples of the skin before skin preparation, after skin preparation and after skin closure were used
Ferro *et al.* (24)	Articular	Three culture samples from the joint capsule, acetabular bone and femoral bone
Font-Vizcarra *et al.* (25)	Articular	Synovial fluid puncture, joint capsule tissue sample, swab of articular surface
Font-Vizcarra *et al.* (26)	Articular	Intra-articular hematoma, capsule tissue sample
Gates *et al.* (27)	Periarticular, articular	Tissue samples from subdeltoid space, tissue from the greater tubercle, tissue samples from deep capsular layer
Grewal *et al.* (28)	Skin, articular	Culture swab along line of skin incision, culture dermis, culture intra-articular humeral head
Haenle *et al.* (29)	Articular	Synovial fluid, joint capsule
Hanada *et al.* (30)	Skin	Swab edge skin incision before closure
James & Gower (31)	Articular	Swab and bone chips femoral head
Jonsson *et al.* (32)	Periarticular, articular	THA: samples from the acetabulum before and after cementing the cup, samples from the fascia after its closure
		TKA: samples from the intercondylar notch before and after cementing, samples from the capsule after its closure
Justesen *et al.* (33)	Skin	Two swabs from wound edge, the first just after incision and the second swab just prior to closure of the skin
Kim *et al.* (34)	Soft tissue	Swabs from dermis (excluded surgical gloves and air)
Knobben *et al.* (35)	Unknown, articular	Swabs from instruments, samples from removed bone
Pattyn *et al.* (36)	Articular	Intracapsular synovial biopsy was taken from the superolateral aspect of the femoral neck
Phuong *et al.* (37)	Articular	Cancellous bone of the femoral head and two femoral head inner and surface swabs
Rattanaprichavej *et al.* (38)	Soft tissue, periarticular, articular	3–5 specimens of burned necrotic tissue from the diathermic knife were randomly collected in the surgical field
Rivera *et al.* (39)	Soft tissue, articular	TKA: two tissue samples from around the femur, two from around the tibia and one from the subcutaneous tissue
		THA: two tissue samples were around the acetabulum, two from around the femur and one from the subcutaneous tissue
Sommerville *et al.* (40)	Articular	Surface swab femoral head, culture swab acetabulum; two tissue samples from the femoral head and hip joint capsule
Symonds *et al.* (41)	Skin, soft tissue, articular	Skin, dermis and tissue from glenohumeral joint
Ibrahim *et al.* (42)	Articular	Swab and bone chip femoral head
Torchia *et al.* (43)	Articular	Synovial fluid, three intra-articular tissue samples, swab distal femur
Torrens *et al.* (44)	Skin, soft tissue, periarticular	Skin, subcutaneous tissue, bursa greater tuberosity, long biceps
Zmistowski *et al.* (45)	Articular	Tissue samples from capsule, glenoid labrum, humeral canal and humeral head

THA, total hip arthroplasty; TKA, total knee arthroplasty.

### Antibiotic prophylaxis

In 22 (85%) studies, the use of antibiotic prophylaxis was reported to be administered at least within 1 h prior to surgery (10 studies within 60 min, 6 studies within 30 min, 5 studies during anesthesia, 1 study at incision and 4 studies unknown). Cefazolin was used in 13 of these studies, while cefuroxime was used in seven studies. The remaining four studies did not specify the type of antibiotic prophylaxis that was used.

### Intraoperative cultures

Details on the intraoperative cultures are reported in Table 3. The overall incidence of contamination was 24.7% (95% CI: 19–30%). Heterogeneity between studies was large (*I*^2^ was 97%). When stratified by anatomical area of the implant, the overall incidence of bacterial contamination for THA was 23.9% (95% CI: 14–34%), for TKA was 18.0% (95% CI: 6–30%), for TSA was 26.7% (95% CI: 18–35%) and for TKA and THA combination was 37.0% (95% CI: 23–51%).

**Table 4:** Quality assessment of the studies by the Newcastle–Ottawa scale.

Study	Selection criteria				Comparability^†^	Outcome criteria			Total (9/9)
	1	2	3	4		1	2	3	
Byrne *et al.* (5)	*	*	*	*	0	*	*	*	7/9
Chalmers *et al.* (21)	*	*	*	*	*	*	*	*	8/9
Chen *et al.* (22)	*	*	*	*	0	*	0	*	6/9
Droll *et al.* (23)	*	*	*	*	*	*	0	*	7/9
Ferro *et al.* (24)	*	*	*	*	0	*	*	*	7/9
Font-Vizcarra *et al.* (25)	*	*	*	*	**	*	*	*	9/9
Font-Vizcarra *et al.* (26)	*	*	*	*	0	*	0	*	6/9
Gates *et al.* (27)	*	*	*	0	**	*	*	*	8/9
Grewal *et al.* (28)	*	*	*	*	**	*	0	0	7/9
Haenle *et al.* (29)	*	*	*	*	0	*	*	*	7/9
Hanada *et al.* (30)	*	*	*	*	*	*	0	0	6/9
James & Gower (31)	*	*	*	*	**	*	*	0	8/9
Jonsson *et al.* (32)	*	*	*	*	0	*	*	*	7/9
Kim *et al.* (34)	*	*	*	*	*	*	*	*	8/9
Knobben *et al.* (35)	*	*	*	*	**	*	*	*	9/9
Pattyn *et al.* (36)	*	*	*	*	0	*	*	*	7/9
Phuong *et al.* (37)	*	*	*	*	0	*	*	*	7/9
Rattanaprichavej *et al.* (38)	*	*	*	*	0	*	*	*	7/9
Rivera *et al.* (39)	*	*	*	*	**	*	*	*	9/9
Sommerville *et al.* (40)	*	*	*	*	0	*	*	*	7/9
Symonds *et al.* (41)	*	*	*	*	*	*	*	*	8/9
Ibrahim *et al.* (42)	*	*	*	0	0	0	*	0	4/9
Justesen*et al.* (33)	*	*	*	*	**	*	*	*	9/9
Torchia *et al.* (43)	*	*	*	*	0	*	*	*	7/9
Torrens *et al.* (44)	*	*	*	*	0	*	*	*	7/9
Zmistowski *et al.* (45)	*	*	*	*	0	*	*	*	7/9

Selection criteria: 1 – representativeness of the exposed cohort; 2 – selection of the non-exposed cohort; 3 – ascertainment exposure; 4 – outcome not present at the start of study. Outcome criteria: 1 – assessment of outcome; 2 – sufficient follow-up time; 3 – adequacy of follow-up.

^†^ Comparability of cohorts (0–2 points).

### Contamination and prosthetic joint infection

**Figure 2:**
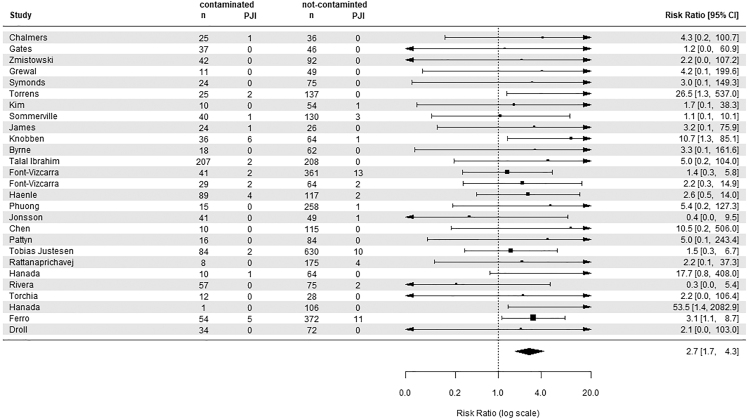
Forest plot showing the risk ratio in prosthetic joint infection between contaminated and non-contaminated arthroplasties.

**Figure 3:**
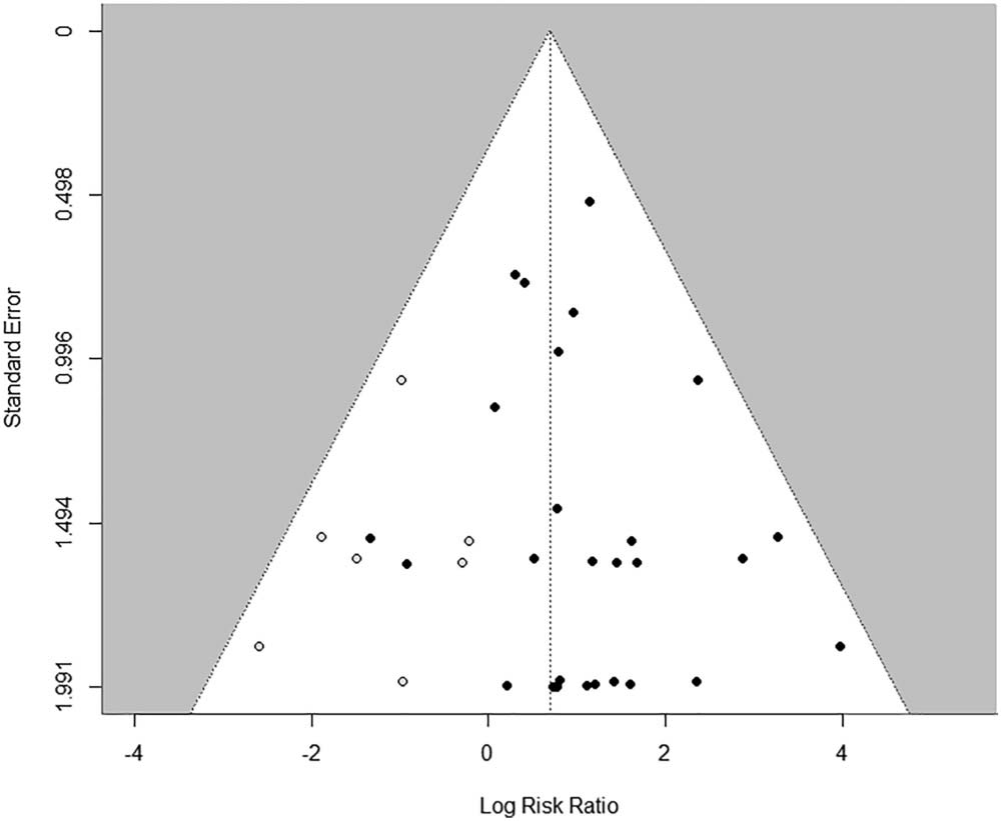
Funnel plot (trim-fitted) included studies for the primary outcome.

The incidence of PJIs was higher in the contaminated group (2.0%; 95% CI: 1.1–2.8%) compared to the group without contamination (1.0%; 0.6–1.4%), with a pooled risk ratio (RR) of 2.7 (95% CI: 1.7–4.3) and no observed heterogeneity among the studies (*I*^2^ = 0%); see Fig. 2. However, the funnel plot (Fig. 3) was asymmetrical, suggesting publication bias. To address this, the trim-and-fill method was employed, which estimated seven missing studies, and after adjustment for this possible publication bias, the RR was 2.0 (95% CI: 1.3–3.1), with no significant heterogeneity observed (*I*^2^ = 0%).

### Matching of microorganisms

Twelve (46%) studies reported on matching the microorganisms cultured during PJIs and the bacterial contaminant during primary joint arthroplasty. In 30% (95% CI: 13–48%) of the contaminated primary joint arthroplasties, the same microorganism was cultured as a cause of PJIs. In the remaining 70% of PJIs, the PJI-cultured microorganisms were different. Within the 30% group of patients with matching between contaminant and PJI microorganisms, differences existed among different types of arthroplasty: 61% of shoulder arthroplasty PJI (95% CI: 19–100%) microorganisms matched the contaminant microorganisms and 22% of lower extremity PJI (95% CI: 6–38) microorganisms matched the contaminant microorganisms.

### Wound complications

Four studies reported postoperative wound complications, such as delayed wound healing or prolonged wound leakage. The pooled comparison of wound complications resulted in an RR of 1.97, with a broad 95% CI (0.54–71) due to the small groups and low incidence of wound complications.

### Contamination and the risk of revision

Fourteen (54%) studies had data on revision surgery. The reason for revision was stratified into septic or aseptic. Revision surgery was done in 32 (3.2%) arthroplasties (6 septic vs 26 aseptic) in the contaminated joint arthroplasties and 44 patients (1.2%, 14 septic vs 30 aseptic) in the non-contaminated group.

**Figure 4:**
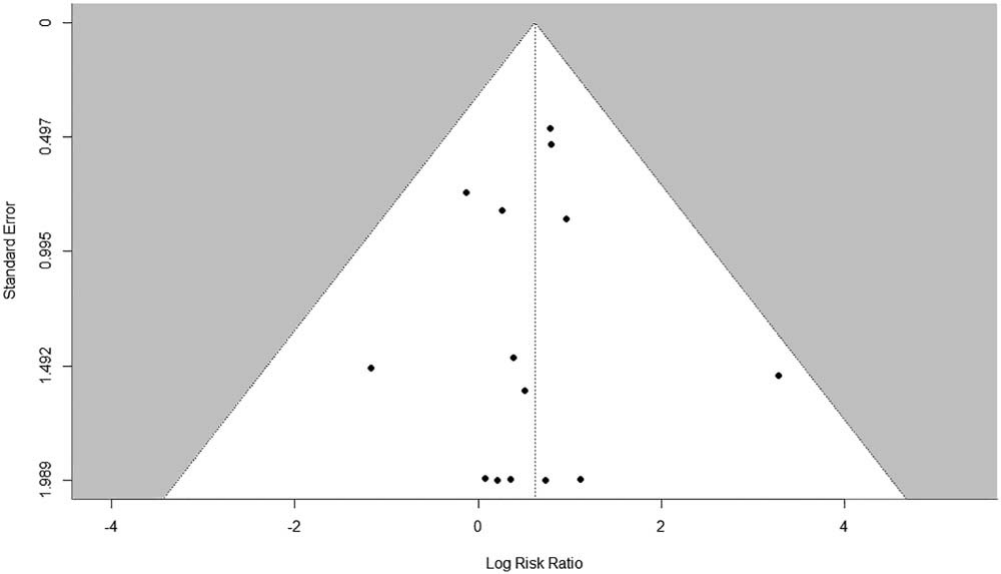
Funnel plot included studies for outcome: revision any reason.

The pooled analysis for risk of revision for any reason in contaminated joint arthroplasties was a mean RR of 1.86 (95% CI: 1.14–3.03). The association between contamination and the risk of aseptic revision (14 studies) was a mean RR of 1.96 (95% CI: 1.13–3.41). The mean RR for revision for septic reasons was 1.82 (95% CI: 0.83–4.00). An examination of the funnel plot (Fig. 4) revealed no evidence of asymmetry.

### Quality assessment

Of the included studies, 22 were classified as high-quality observational studies based on the Newcastle–Ottawa scale, while four studies were classified as moderate quality. The average quality assessment score across all included studies is 7.2. A detailed breakdown of the Newcastle–Ottawa scale scores for each study is presented in Table 4.

## Discussion

### Main results

The purpose of this systematic review and meta-analysis was to evaluate the relationship between detectable perioperative contamination during primary joint arthroplasty and the subsequent development of prosthetic joint infections. Our findings indicate that culture-proven intraoperative contamination is associated with an increased risk of developing PJIs with a pooled adjusted RR of 2.0 (95% CI: 1.3–3.1). Despite extensive measures implemented by surgeons to minimize the risk of intraoperative contamination, such as laminal flow and antimicrobial incision drapes, we observed a relatively high contamination rate of 24.7%. During the joint arthroplasty surgery, small numbers of bacteria from the skin, surgical instruments, air or surrounding tissues can enter the surgical site and potentially form a biofilm, leading to PJIs. Detecting minimal contamination is difficult, as conventional culture methods have limited sensitivity when there are low bacterial loads (10). Therefore, the various sampling techniques, such as wound swabs or tissue samples, may underestimate the true contamination. This likely represents a misclassification problem, where some true contaminations remain undetected and are classified as non-contaminated. Fortunately, the contamination resulted in a prosthetic joint infection rate of only 2.0% in the contaminated group with the same microorganism cultured in only 30% of the contaminated primary joint arthroplasties with PJIs. This suggests that in more than nine out of ten cases of contaminated joint arthroplasties, the host’s immune response was able to clear the microorganism without progressing to PJIs.

Regarding revision surgery, an increased risk of revision surgery was observed in patients with contaminated joint arthroplasty based on 14 observational cohort studies. The risk of aseptic revision, with a pooled RR of 1.96 (95% CI: 1.13–3.41), was comparable to the risk of septic revision, with a pooled RR of 1.82 (95% CI: 0.83–4.00), with more uncertainty in septic revisions. These findings might contradict the previous understanding of contamination and periprosthetic joint infection. Sundfelt *et al.* published an interesting theory describing the effect of endotoxins on prostheses and the immune response. While bacteria introduced during contamination might be eradicated by antibiotic prophylaxis and the host immune system, remnants of the bacterial membrane may still be present on the prosthesis. The residual material may trigger a macrophage activation and osteolysis, leading to ‘aseptic loosening’ of the prosthesis (11). Consequently, even in patients who successfully clear bacterial contamination during primary surgery, there may be a greater risk of long-term complications, such as loosening of the prosthesis, resulting in revision surgery. Alternatively, loosening of prosthesis could result from a culture-negative PJI with a low virulent microorganism and, therefore, misdiagnosed for aseptic revision surgery.

Regarding the differences between the two groups, the contaminated group was on average 1.3 years younger than the non-contaminated group. There were fewer females in the contaminated group across all arthroplasties. However, this difference was not observed when focusing specifically on hip and knee arthroplasties. Otherwise, the groups were on baseline the same, suggesting that contamination is not related to these risk factors for PJIs. Hence, contamination is an independent, but relatively small, risk factor.

### Pathways to prosthetic joint infection and review of the literature

Recent literature has proposed a new pathway for the development of SSIs and PJIs, called the Trojan horse hypothesis. This hypothesis suggests that microorganisms from distant sites, such as the oral cavity and gastrointestinal tract, travel to the wound site through uptake by macrophages and neutrophils, where they cause an infection without detectable bacteremia (2). This hypothesis was tested by Zhu *et al.* (12), who investigated how intestinal methicillin-resistant *Staphylococcus aureus* (MRSA) colonization can lead to prosthetic joint infections (PJIs) after arthroplasty in rats. They proposed the ‘Trojan horse’ mechanism, where MRSA inside neutrophils travel from the gut to the surgical site, causing PJIs. In experiments with rats, they found that intestinal MRSA colonization can cause PJIs and neutrophils carrying MRSA play a crucial role. The study emphasized the importance of considering gut colonization as a potential source for PJIs. In addition, a similar study by Krezalek *et al.* investigated whether MRSA in the intestines could cause SSIs by having immune cells transport MRSA from the gut to surgical wounds (13). The researchers used mice, colonized their guts with MRSA after disrupting gut bacteria with antibiotics and then performed surgical injury and rectus muscle injury. Wound cultures were taken before closure, all negative, ruling out contamination. Some mice developed visible abscesses, while others had MRSA in rectus muscle cultures without visible abscesses. Injecting MRSA into the bloodstream did not cause SSIs. Flow cytometry showed circulating neutrophils carrying MRSA, supporting the idea that immune cells could transfer gut-derived MRSA to wounds as a potential cause of SSIs. This suggests that it is plausible that immune cells act as Trojan horses for gastrointestinal bacteria to cause SSIs in mice. Further research in humans is needed to determine whether this explains why (prosthetic joint) infections can occur without intraoperative contamination yet develop later in the postoperative period.

**Figure 5:**
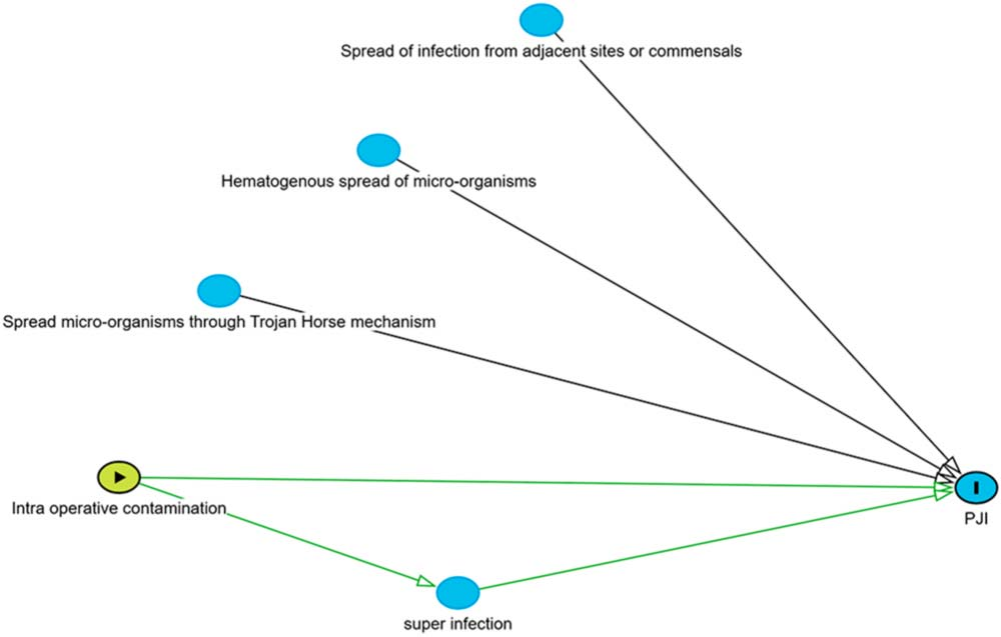
Pathways prosthetic joint infection.

Furthermore, bacteria also present at the surgical site before surgery, known as commensals, can play a role in the development of PJIs. Studies have shown multiple microbiomes for native hip, knee and shoulder joints by taking aspirates and cultures (14, 15, 16). After surgery, there is a local inflammation reaction via migrating inflammatory cells, caused by surgical tissue insult. This is normally accompanied by a systemic inflammatory response characterized by an elevated body temperature and increased levels of inflammatory cytokines (17). Therefore, we believe that the integrity of a wound is very important in the process of wound healing and development of a prosthetic joint infection. A compromised wound and impaired local immune response could lead to a ‘superinfection’ of the wound site by being unable to clear certain organisms postoperatively; see Fig. 5. Subsequently, this leads to an infection of the prosthesis site, whether it is by secondary wound infection of skin commensals or through the Trojan horse mechanism. This would rationalize why patients with risk factors for a compromised wound and impaired immune response (such as diabetes, obesity, active smoking status, rheumatoid arthritis and the use of immunosuppressive and anticoagulant drugs (18)) are more susceptible to developing a PJI after arthroplasty.

In a systematic review and meta-analysis by Ailaney *et al.*, the effect of closed incision negative pressure wound therapy (ciNPWT) compared to standard wound dressing after primary and revision THA and TKA was evaluated (19). Their analysis concluded that the use of ciNPWT after revision surgery reduced the risk of developing SSIs. However, when primary total hip and knee surgery was considered, no significant difference was found between the use of standard wound dressings and ciNPWT (19). Negative pressure wound therapy provides a sterile environment for the wound and prevents postoperative external wound contamination by reducing exudate, modulating inflammation, facilitating immune responders and increasing lymphatic drainage (19). This may help prevent the development of a ‘superinfection’ of the surgical site. When considering revision surgery, soft tissues and vascular structures may be compromised by repetitive surgical damage, resulting in suboptimal wound conditions and immune responses. This could support the concept of patients with compromised wounds/soft tissues having a greater risk of developing an infection, regardless of intraoperative contamination.

### Strengths and limitations

After the selection process, we conducted a reference search to ensure that no relevant studies were missed. This allowed us to access a large population group and pool the data for a meta-analysis of this relatively rare complication. In addition, most of our included studies were of high quality (*n* = 22) and the remaining studies (*n* = 4) were of moderate quality.

The following limitations should be taken into account when interpreting our results.

First, there is a possibility that surgeons may have failed to detect bacteria or other microorganisms when collecting swabs or tissue samples during the primary arthroplasty. This could result in false-negative interpretations when assessing perioperative contamination. Thus, this systematic review focuses on detectable contamination. The included studies used different definitions and sample sites to test intraoperative contamination, such as skin swabs, synovial fluid and tissue/deep samples. Due to inconsistent reporting, stratified analysis by sample site was not viable. Future studies should implement a more standardized technique to improve comparability. Second, the studies did not measure the total bacterial load when taking cultures in patients undergoing primary joint arthroplasty. It is possible that the amount of bacterial load may correlate with the risk of developing PJIs, assuming a patient is able to clear few bacteria from the surgical site compared to a point where the bacterial load exceeds a certain threshold where the immune system of a patient or the perioperative antibiotic prophylaxis is unable to clear the bacteria. Third, in case of matching microorganisms between contamination and clinically evident PJIs, no genotyping was done to ensure it was the same strain from a single species. Especially in shoulder arthroplasty, where *Cutibacterium acnes* is common and even a commensal, this could be relevant (20). In our meta-analysis, only 12 studies performed microorganism matching between intraoperative contamination and subsequent PJIs, and only in 30% of the cases with PJIs, the same microorganism was found. This limited overlap may suggest that many intraoperative positive cultures may represent clinically irrelevant contamination or supports the concept of a superinfection by another (more virulent) microorganism in an already compromised surgical site. Future studies should systematically report the matching of microorganisms to better clarify the clinical relevance of intraoperative contamination. Fourth, only 14 (54%) studies reported a follow-up time longer than 1 year. Incidentally, chronic PJIs may manifest beyond this period and revision surgery may be performed after the first year. The limited follow-up time may have led to a slight underestimation of the true complication rate.

Finally, the lack of consistent reporting of patient comorbidities (such as diabetes mellitus and smoking), medication use, postoperative wound problems or details on the surgery (such as duration of surgery) across the included studies ensured that there were not enough data for a comprehensive meta-analysis of these factors.

## Conclusion

The incidence of detectable intraoperative contamination was 24.7%. Intraoperative contamination increased the risk of PJIs by a factor of 2.0, revision for any reason by a factor of 1.86 and revision for aseptic loosening by a factor of 1.96. While the relative risk was increased, the absolute risk of complications was relatively low after contamination: more than nine out of ten patients with a contaminated joint arthroplasty did not develop any complications attributed to the contamination itself.

## Supplementary materials



## ICMJE Statement of Interest

The authors declare that there is no conflict of interest that could be perceived as prejudicing the impartiality of the work reported.

## Funding Statement

This work did not receive any specific grant from any funding agency in the public, commercial or not-for-profit sector.

## Data availability

The data are available upon reasonable request by contacting the corresponding author.
