# Supplementary material for: The incidence of detectable bacterial contaminated primary joint arthroplasties and periprosthetic joint infection: a systematic review and meta-analysis
Source: EFORT Open Rev. 2026 Jun 1;11(6):524–35. doi: 10.1530/EOR-2025-0074 (PMC13238974; doi:10.1530/EOR-2025-0074)
Supplement: Supplementary file 1 [file supplementary_materials.pdf]

Supplement file – search strategy

PubMed

(("perioperative contamination"[tw] OR "perioperative contaminations"[tw] OR "perioperative contaminat*"[tw] OR "peri operative contamination"[tw] OR "peri operative contaminat*"[tw] OR "intraoperative contamination"[tw] OR "intraoperative contaminat*"[tw] OR "intra operative contamination"[tw] OR "intra operative contaminat*"[tw] OR "perioperative bacterial contamination"[tw] OR "perioperative bacterial contaminat*"[tw] OR "intraoperative bacterial contamination"[tw] OR "intraoperative bacterial contaminat*"[tw] OR "intra operative bacterial contamination"[tw] OR "intra operative bacterial contaminat*"[tw] OR "positive intraoperative cultures"[tw] OR "positive intraoperative culture"[tw] OR "positive intra operative cultures"[tw] OR "perioperative cultures"[tw] OR "perioperative cultures"[tw] OR "intraoperative cultures"[tw] OR "intraoperative culture"[tw] OR "intra operative cultures"[tw] OR "intra operative culture"[tw] OR "intraoperative bacterial cultures"[tw] OR "intraoperative bacterial culture"[tw] OR "intra operative bacterial cultures"[tw] OR "intraoperative bacterial cultures"[tw] OR "intraoperative bacterial culture"[tw] OR "intra operative bacterial cultures"[tw] OR "peroperative contamination"[tw] OR "peroperative contaminat*"[tw] OR "peroperative bacterial contamination"[tw] OR "peroperative bacterial contaminat*"[tw] OR "peroperative cultures"[tw] OR "peroperative cultur*"[tw] OR "per operative culture"[tw] OR "per operative cultures"[tw] OR "per operative cultur*"[tw] OR (("culture"[tw] OR "cultures"[tw] OR "cultured"[tw]) AND ("contamination"[tw] OR "contaminated"[tw] OR "contaminate"[tw] OR "contaminat*"[tw] OR "sample"[tw] OR "samples"[tw]) AND ("Perioperative Period"[Mesh] OR "Intraoperative Period"[Mesh] OR "intraoperative"[tw] OR "intra operative"[tw] OR "perioperative"[tw] OR "peri operative"[tw] OR "peroperative"[tw] OR "per operative"[tw] OR "during surgery"[tw] OR "during surg*"[tw] OR "during"[tw] OR "Surgical Wound Infection"[Mesh])) OR (("culture positive"[tw] OR "culture positiv*"[tw] OR "cultures positive"[tw] OR "cultured positive"[tw] OR "positive culture"[tw] OR "positive cultures"[tw] OR "positive cultur*"[tw]) AND ("Perioperative Period"[Mesh] OR "Intraoperative Period"[Mesh] OR "intraoperative"[tw] OR "intra operative"[tw] OR "perioperative"[tw] OR "peri operative"[tw] OR "peroperative"[tw] OR "per operative"[tw] OR "during surgery"[tw] OR "during surg*"[tw] OR "during"[tw] OR "Surgical Wound Infection"[Mesh])) OR "wound contamination"[tw] OR "wound contaminat*"[tw] OR "wounds contaminated"[tw] OR "wound contaminat*"[tw]) AND ("Prosthesis related infections"[mesh] OR "prosthesis related infections"[tw] OR "Prosthesis Related Infection"[tw] OR "Prosthesis Infections"[tw] OR "Prosthesis Infection"[tw] OR "Prosthetic Infections"[tw] OR "Prosthetic Infection"[tw] OR "Periprosthesis Infections"[tw] OR "Periprosthetic Infections"[tw] OR "Periprosthetic Infection"[tw] OR "Prosthetic joint Infections"[tw] OR "Prosthetic joint Infection"[tw] OR "Periprosthetic joint Infections"[tw] OR "Periprosthetic joint Infection"[tw] OR "Prosthetic knee Infections"[tw] OR "Prosthetic knee Infection"[tw] OR "Periprosthetic knee Infections"[tw] OR "Periprosthetic knee Infection"[tw] OR "Prosthetic hip Infections"[tw] OR "Prosthetic hip Infection"[tw] OR "Periprosthetic hip Infections"[tw] OR "Periprosthetic hip Infection"[tw] OR "Prosthetic shoulder Infections"[tw] OR "Prosthetic shoulder Infection"[tw] OR "Periprosthetic shoulder Infections"[tw] OR "Periprosthetic shoulder Infection"[tw] OR "Prosthetic elbow Infection"[tw] OR "Periprosthetic elbow Infection"[tw] OR "Periprosthesis Infect*"[tw] OR

"Periprosthetic elbow Infect*"[tw] OR "Periprosthetic hip Infect*"[tw] OR "Periprosthetic Infect*"[tw] OR "Periprosthetic joint Infect*"[tw] OR "Periprosthetic knee Infect*"[tw] OR "Periprosthetic shoulder Infect*"[tw] OR "Prosthesis Infect*"[tw] OR "Prosthetic elbow Infect*"[tw] OR "Prosthetic hip Infect*"[tw] OR "Prosthetic Infect*"[tw] OR "Prosthetic joint Infect*"[tw] OR "Prosthetic knee Infect*"[tw] OR "Prosthetic shoulder Infect*"[tw]))

MEDLINE (OVID)

(("perioperative contamination".mp OR "perioperative contaminations".mp OR "perioperative contaminat*".mp OR "peri operative contamination".mp OR "peri operative contaminat*".mp OR "intraoperative contamination".mp OR "intraoperative contaminat*".mp OR "intra operative contamination".mp OR "intra operative contaminat*".mp OR "perioperative bacterial contamination".mp OR "perioperative bacterial contaminat*".mp OR "intraoperative bacterial contamination".mp OR "intraoperative bacterial contaminat*".mp OR "intra operative bacterial contamination".mp OR "intra operative bacterial contaminat*".mp OR "positive intraoperative cultures".mp OR "positive intraoperative culture".mp OR "positive intra operative cultures".mp OR "perioperative cultures".mp OR "perioperative cultures".mp OR "intraoperative cultures".mp OR "intraoperative culture".mp OR "intra operative cultures".mp OR "intra operative culture".mp OR "intraoperative bacterial cultures".mp OR "intraoperative bacterial culture".mp OR "intra operative bacterial cultures".mp OR "intraoperative bacterial cultures".mp OR "intraoperative bacterial culture".mp OR "intra operative bacterial cultures".mp OR "peroperative contamination".mp OR "peroperative contaminat*".mp OR "peroperative bacterial contamination".mp OR "peroperative bacterial contaminat*".mp OR "peroperative cultures".mp OR "peroperative cultur*".mp OR "per operative culture".mp OR "per operative cultures".mp OR "per operative cultur*".mp OR (("culture".mp OR "cultures".mp OR "cultured".mp) AND ("contamination".mp OR "contaminated".mp OR "contaminate".mp OR "contaminat*".mp OR "sample".mp OR "samples".mp) AND (exp "Perioperative Period"/ OR exp "Intraoperative Period"/ OR "intraoperative".mp OR "intra operative".mp OR "perioperative".mp OR "peri operative".mp OR "peroperative".mp OR "per operative".mp OR "during surgery".mp OR "during surg*".mp OR "during".mp OR exp "Surgical Wound Infection"/)) OR (("culture positive".mp OR "culture positiv*".mp OR "cultures positive".mp OR "cultured positive".mp OR "positive culture".mp OR "positive cultures".mp OR "positive cultur*".mp) AND (exp "Perioperative Period"/ OR exp "Intraoperative Period"/ OR "intraoperative".mp OR "intra operative".mp OR "perioperative".mp OR "peri operative".mp OR "peroperative".mp OR "per operative".mp OR "during surgery".mp OR "during surg*".mp OR "during".mp OR exp "Surgical Wound Infection"/)) OR "wound contamination".mp OR "wound contaminat*".mp OR "wounds contaminated".mp OR "wound contaminat*".mp) AND (exp "Prosthesis related infections"/ OR "prosthesis related infections".mp OR "Prosthesis Related Infection".mp OR "Prosthesis Infections".mp OR "Prosthesis Infection".mp OR "Prosthetic Infections".mp OR "Prosthetic Infection".mp OR "Periprosthesis Infections".mp OR "Periprosthetic Infections".mp OR "Periprosthetic Infection".mp OR "Prosthetic joint Infections".mp OR "Prosthetic joint Infection".mp OR "Periprosthetic joint Infections".mp OR "Periprosthetic joint Infection".mp OR "Prosthetic knee Infections".mp OR "Prosthetic knee Infection".mp OR "Periprosthetic knee Infections".mp OR "Periprosthetic knee Infection".mp OR "Prosthetic hip Infections".mp OR "Prosthetic hip Infection".mp OR "Periprosthetic hip Infections".mp OR

"Periprosthetic hip Infection".mp OR "Prosthetic shoulder Infections".mp OR "Prosthetic shoulder Infection".mp OR "Periprosthetic shoulder Infections".mp OR "Periprosthetic shoulder Infection".mp OR "Prosthetic elbow Infection".mp OR "Periprosthetic elbow Infection".mp OR "Periprosthesis Infect*".mp OR "Periprosthetic elbow Infect*".mp OR "Periprosthetic hip Infect*".mp OR "Periprosthetic Infect*".mp OR "Periprosthetic joint Infect*".mp OR "Periprosthetic knee Infect*".mp OR "Periprosthetic shoulder Infect*".mp OR "Prosthesis Infect*".mp OR "Prosthetic elbow Infect*".mp OR "Prosthetic hip Infect*".mp OR "Prosthetic Infect*".mp OR "Prosthetic joint Infect*".mp OR "Prosthetic knee Infect*".mp OR "Prosthetic shoulder Infect*".mp))

Embase

(("perioperative contamination".mp OR "perioperative contaminations".mp OR "perioperative contaminat*".mp OR "peri operative contamination".mp OR "peri operative contaminat*".mp OR "intraoperative contamination".mp OR "intraoperative contaminat*".mp OR "intra operative contamination".mp OR "intra operative contaminat*".mp OR "perioperative bacterial contamination".mp OR "perioperative bacterial contaminat*".mp OR "intraoperative bacterial contamination".mp OR "intraoperative bacterial contaminat*".mp OR "intra operative bacterial contamination".mp OR "intra operative bacterial contaminat*".mp OR "positive intraoperative cultures".mp OR "positive intraoperative culture".mp OR "positive intra operative cultures".mp OR "perioperative cultures".mp OR "perioperative cultures".mp OR "intraoperative cultures".mp OR "intraoperative culture".mp OR "intra operative cultures".mp OR "intra operative culture".mp OR "intraoperative bacterial cultures".mp OR "intraoperative bacterial culture".mp OR "intra operative bacterial cultures".mp OR "intraoperative bacterial cultures".mp OR "intraoperative bacterial culture".mp OR "intra operative bacterial cultures".mp OR "peroperative contamination".mp OR "peroperative contaminat*".mp OR "peroperative bacterial contamination".mp OR "peroperative bacterial contaminat*".mp OR "peroperative cultures".mp OR "peroperative cultur*".mp OR "per operative culture".mp OR "per operative cultures".mp OR "per operative cultur*".mp OR ((exp "bacterium culture"/ OR "culture".mp OR "cultures".mp OR "cultured".mp) AND (exp "microbial contamination"/ OR "contamination".mp OR "contaminated".mp OR "contaminate".mp OR "contaminat*".mp OR "sample".mp OR "samples".mp) AND (exp "perioperative period"/ OR exp "intraoperative period"/ OR "intraoperative".mp OR "intra operative".mp OR "perioperative".mp OR "peri operative".mp OR "peroperative".mp OR "per operative".mp OR "during surgery".mp OR "during surg*".mp OR "during".mp OR exp "Wound Infection"/)) OR (("culture positive".mp OR "culture positiv*".mp OR "cultures positive".mp OR "cultured positive".mp OR "positive culture".mp OR "positive cultures".mp OR "positive cultur*".mp) AND (exp "Perioperative Period"/ OR exp "Intraoperative Period"/ OR "intraoperative".mp OR "intra operative".mp OR "perioperative".mp OR "peri operative".mp OR "peroperative".mp OR "per operative".mp OR "during surgery".mp OR "during surg*".mp OR "during".mp OR "Surgical Wound Infection"/)) OR "wound contamination".mp OR "wound contaminat*".mp OR "wounds contaminated".mp OR "wound contaminat*".mp) AND (exp "Prosthesis infection"/ OR "prosthesis related infections".mp OR "Prosthesis Related Infection".mp OR "Prosthesis Infections".mp OR "Prosthesis Infection".mp OR "Prosthetic Infections".mp OR "Prosthetic Infection".mp OR "Periprosthesis Infections".mp OR "Periprosthetic Infections".mp OR "Periprosthetic Infection".mp OR "Prosthetic joint

Infections".mp OR "Prosthetic joint Infection".mp OR "Periprosthetic joint Infections".mp OR "Periprosthetic joint Infection".mp OR "Prosthetic knee Infections".mp OR "Prosthetic knee Infection".mp OR "Periprosthetic knee Infections".mp OR "Periprosthetic knee Infection".mp OR "Prosthetic hip Infections".mp OR "Prosthetic hip Infection".mp OR "Periprosthetic hip Infections".mp OR "Periprosthetic hip Infection".mp OR "Prosthetic shoulder Infections".mp OR "Prosthetic shoulder Infection".mp OR "Periprosthetic shoulder Infections".mp OR "Periprosthetic shoulder Infection".mp OR "Prosthetic elbow Infection".mp OR "Periprosthetic elbow Infection".mp OR "Periprosthesis Infect*".mp OR "Periprosthetic elbow Infect*".mp OR "Periprosthetic hip Infect*".mp OR "Periprosthetic Infect*".mp OR "Periprosthetic joint Infect*".mp OR "Periprosthetic knee Infect*".mp OR "Periprosthetic shoulder Infect*".mp OR "Prosthesis Infect*".mp OR "Prosthetic elbow Infect*".mp OR "Prosthetic hip Infect*".mp OR "Prosthetic Infect*".mp OR "Prosthetic joint Infect*".mp OR "Prosthetic knee Infect*".mp OR "Prosthetic shoulder Infect*".mp)) NOT (conference review or conference abstract).pt

Web of Science

TS=(("perioperative contamination" OR "perioperative contaminations" OR "perioperative contaminat*" OR "peri operative contamination" OR "peri operative contaminat*" OR "intraoperative contamination" OR "intraoperative contaminat*" OR "intra operative contamination" OR "intra operative contaminat*" OR "perioperative bacterial contamination" OR "perioperative bacterial contaminat*" OR "intraoperative bacterial contamination" OR "intraoperative bacterial contaminat*" OR "intra operative bacterial contamination" OR "intra operative bacterial contaminat*" OR "positive intraoperative cultures" OR "positive intraoperative culture" OR "positive intra operative cultures" OR "perioperative cultures" OR "perioperative cultures" OR "intraoperative cultures" OR "intraoperative culture" OR "intra operative cultures" OR "intra operative culture" OR "intraoperative bacterial cultures" OR "intraoperative bacterial culture" OR "intra operative bacterial cultures" OR "intraoperative bacterial cultures" OR "intraoperative bacterial culture" OR "intra operative bacterial cultures" OR "peroperative contamination" OR "peroperative contaminat*" OR "peroperative bacterial contamination" OR "peroperative bacterial contaminat*" OR "peroperative cultures" OR "peroperative cultur*" OR "per operative culture" OR "per operative cultures" OR "per operative cultur*" OR (("bacterium culture" OR "culture" OR "cultures" OR "cultured") AND ("microbial contamination" OR "contamination" OR "contaminated" OR "contaminate" OR "contaminat*" OR "sample" OR "samples") AND ("perioperative period" OR "intraoperative period" OR "intraoperative" OR "intra operative" OR "perioperative" OR "peri operative" OR "peroperative" OR "per operative" OR "during surgery" OR "during surg*" OR "during" OR "Surgical Wound Infection")) OR (("culture positive" OR "culture positiv*" OR "cultures positive" OR "cultured positive" OR "positive culture" OR "positive cultures" OR "positive cultur*") AND ("Perioperative Period" OR "Intraoperative Period" OR "intraoperative" OR "intra operative" OR "perioperative" OR "peri operative" OR "peroperative" OR "per operative" OR "during surgery" OR "during surg*" OR "during" OR "Surgical Wound Infection")) OR "wound contamination" OR "wound contaminat*" OR "wounds contaminated" OR "wound contaminat*") AND ("Prosthesis infection" OR "prosthesis related infections" OR "Prosthesis Related Infection" OR "Prosthesis Infections" OR "Prosthesis Infection" OR "Prosthetic

Infections" OR "Prosthetic Infection" OR "Periprosthesis Infections" OR "Periprosthetic Infections" OR "Periprosthetic Infection" OR "Prosthetic joint Infections" OR "Prosthetic joint Infection" OR "Periprosthetic joint Infections" OR "Periprosthetic joint Infection" OR "Prosthetic knee Infections" OR "Prosthetic knee Infection" OR "Periprosthetic knee Infections" OR "Periprosthetic knee Infection" OR "Prosthetic hip Infections" OR "Prosthetic hip Infection" OR "Periprosthetic hip Infections" OR "Periprosthetic hip Infection" OR "Prosthetic shoulder Infections" OR "Prosthetic shoulder Infection" OR "Periprosthetic shoulder Infections" OR "Periprosthetic shoulder Infection" OR "Prosthetic elbow Infection" OR "Periprosthetic elbow Infection" OR "Periprosthesis Infect*" OR "Periprosthetic elbow Infect*" OR "Periprosthetic hip Infect*" OR "Periprosthetic Infect*" OR "Periprosthetic joint Infect*" OR "Periprosthetic knee Infect*" OR "Periprosthetic shoulder Infect*" OR "Prosthesis Infect*" OR "Prosthetic elbow Infect*" OR "Prosthetic hip Infect*" OR "Prosthetic Infect*" OR "Prosthetic joint Infect*" OR "Prosthetic knee Infect*" OR "Prosthetic shoulder Infect*")) NOT DT=(meeting abstract)

Cochrane Library

(("perioperative contamination" OR "perioperative contaminations" OR "perioperative contaminat*" OR "peri operative contamination" OR "peri operative contaminat*" OR "intraoperative contamination" OR "intraoperative contaminat*" OR "intra operative contamination" OR "intra operative contaminat*" OR "perioperative bacterial contamination" OR "perioperative bacterial contaminat*" OR "intraoperative bacterial contamination" OR "intraoperative bacterial contaminat*" OR "intra operative bacterial contamination" OR "intra operative bacterial contaminat*" OR "positive intraoperative cultures" OR "positive intraoperative culture" OR "positive intra operative cultures" OR "perioperative cultures" OR "perioperative cultures" OR "intraoperative cultures" OR "intraoperative culture" OR "intra operative cultures" OR "intra operative culture" OR "intraoperative bacterial cultures" OR "intraoperative bacterial culture" OR "intra operative bacterial cultures" OR "intraoperative bacterial cultures" OR "intraoperative bacterial culture" OR "intra operative bacterial cultures" OR "peroperative contamination" OR "peroperative contaminat*" OR "peroperative bacterial contamination" OR "peroperative bacterial contaminat*" OR "peroperative cultures" OR "peroperative cultur*" OR "per operative culture" OR "per operative cultures" OR "per operative cultur*" OR (("bacterium culture" OR "culture" OR "cultures" OR "cultured") AND ("microbial contamination" OR "contamination" OR "contaminated" OR "contaminate" OR "contaminat*" OR "sample" OR "samples") AND ("perioperative period" OR "intraoperative period" OR "intraoperative" OR "intra operative" OR "perioperative" OR "peri operative" OR "peroperative" OR "per operative" OR "during surgery" OR "during surg*" OR "during" OR "Surgical Wound Infection")) OR (("culture positive" OR "culture positiv*" OR "cultures positive" OR "cultured positive" OR "positive culture" OR "positive cultures" OR "positive cultur*") AND ("Perioperative Period" OR "Intraoperative Period" OR "intraoperative" OR "intra operative" OR "perioperative" OR "peri operative" OR "peroperative" OR "per operative" OR "during surgery" OR "during surg*" OR "during" OR "Surgical Wound Infection")) OR "wound contamination" OR "wound contaminat*" OR "wounds contaminated" OR "wound contaminat*") AND ("Prosthesis infection" OR "prosthesis related infections" OR "Prosthesis Related Infection" OR "Prosthesis Infections" OR "Prosthesis Infection" OR "Prosthetic

Infections" OR "Prosthetic Infection" OR "Periprosthesis Infections" OR "Periprosthetic Infections" OR "Periprosthetic Infection" OR "Prosthetic joint Infections" OR "Prosthetic joint Infection" OR "Periprosthetic joint Infections" OR "Periprosthetic joint Infection" OR "Prosthetic knee Infections" OR "Prosthetic knee Infection" OR "Periprosthetic knee Infections" OR "Periprosthetic knee Infection" OR "Prosthetic hip Infections" OR "Prosthetic hip Infection" OR "Periprosthetic hip Infections" OR "Periprosthetic hip Infection" OR "Prosthetic shoulder Infections" OR "Prosthetic shoulder Infection" OR "Periprosthetic shoulder Infections" OR "Periprosthetic shoulder Infection" OR "Prosthetic elbow Infection" OR "Periprosthetic elbow Infection" OR "Periprosthesis Infect*" OR "Periprosthetic elbow Infect*" OR "Periprosthetic hip Infect*" OR "Periprosthetic Infect*" OR "Periprosthetic joint Infect*" OR "Periprosthetic knee Infect*" OR "Periprosthetic shoulder Infect*" OR "Prosthesis Infect*" OR "Prosthetic elbow Infect*" OR "Prosthetic hip Infect*" OR "Prosthetic Infect*" OR "Prosthetic joint Infect*" OR "Prosthetic knee Infect*" OR "Prosthetic shoulder Infect*")):ti,ab,kw

Emcare (OVID)

(("perioperative contamination".mp OR "perioperative contaminations".mp OR "perioperative contaminat*".mp OR "peri operative contamination".mp OR "peri operative contaminat*".mp OR "intraoperative contamination".mp OR "intraoperative contaminat*".mp OR "intra operative contamination".mp OR "intra operative contaminat*".mp OR "perioperative bacterial contamination".mp OR "perioperative bacterial contaminat*".mp OR "intraoperative bacterial contamination".mp OR "intraoperative bacterial contaminat*".mp OR "intra operative bacterial contamination".mp OR "intra operative bacterial contaminat*".mp OR "positive intraoperative cultures".mp OR "positive intraoperative culture".mp OR "positive intra operative cultures".mp OR "perioperative cultures".mp OR "perioperative cultures".mp OR "intraoperative cultures".mp OR "intraoperative culture".mp OR "intra operative cultures".mp OR "intra operative culture".mp OR "intraoperative bacterial cultures".mp OR "intraoperative bacterial culture".mp OR "intra operative bacterial cultures".mp OR "intraoperative bacterial cultures".mp OR "intraoperative bacterial culture".mp OR "intra operative bacterial cultures".mp OR "peroperative contamination".mp OR "peroperative contaminat*".mp OR "peroperative bacterial contamination".mp OR "peroperative bacterial contaminat*".mp OR "peroperative cultures".mp OR "peroperative cultur*".mp OR "per operative culture".mp OR "per operative cultures".mp OR "per operative cultur*".mp OR ((exp "bacterium culture"/ OR "culture".mp OR "cultures".mp OR "cultured".mp) AND (exp "microbial contamination"/ OR "contamination".mp OR "contaminated".mp OR "contaminate".mp OR "contaminat*".mp OR "sample".mp OR "samples".mp) AND (exp "perioperative period"/ OR exp "intraoperative period"/ OR "intraoperative".mp OR "intra operative".mp OR "perioperative".mp OR "peri operative".mp OR "peroperative".mp OR "per operative".mp OR "during surgery".mp OR "during surg*".mp OR "during".mp OR exp "Wound Infection"/)) OR (("culture positive".mp OR "culture positiv*".mp OR "cultures positive".mp OR "cultured positive".mp OR "positive culture".mp OR "positive cultures".mp OR "positive cultur*".mp) AND (exp "Perioperative Period"/ OR exp "Intraoperative Period"/ OR "intraoperative".mp OR "intra operative".mp OR "perioperative".mp OR "peri operative".mp OR "peroperative".mp OR "per operative".mp OR "during surgery".mp OR "during surg*".mp OR

"during".mp OR "Surgical Wound Infection"/)) OR "wound contamination".mp OR "wound contaminat*".mp OR "wounds contaminated".mp OR "wound contaminat*".mp) AND (exp "Prosthesis infection"/ OR "prosthesis related infections".mp OR "Prosthesis Related Infection".mp OR "Prosthesis Infections".mp OR "Prosthesis Infection".mp OR "Prosthetic Infections".mp OR "Prosthetic Infection".mp OR "Periprosthesis Infections".mp OR "Periprosthetic Infections".mp OR "Periprosthetic Infection".mp OR "Prosthetic joint Infections".mp OR "Prosthetic joint Infection".mp OR "Periprosthetic joint Infections".mp OR "Periprosthetic joint Infection".mp OR "Prosthetic knee Infections".mp OR "Prosthetic knee Infection".mp OR "Periprosthetic knee Infections".mp OR "Periprosthetic knee Infection".mp OR "Prosthetic hip Infections".mp OR "Prosthetic hip Infection".mp OR "Periprosthetic hip Infections".mp OR "Periprosthetic hip Infection".mp OR "Prosthetic shoulder Infections".mp OR "Prosthetic shoulder Infection".mp OR "Periprosthetic shoulder Infections".mp OR "Periprosthetic shoulder Infection".mp OR "Prosthetic elbow Infection".mp OR "Periprosthetic elbow Infection".mp OR "Periprosthesis Infect*".mp OR "Periprosthetic elbow Infect*".mp OR "Periprosthetic hip Infect*".mp OR "Periprosthetic Infect*".mp OR "Periprosthetic joint Infect*".mp OR "Periprosthetic knee Infect*".mp OR "Periprosthetic shoulder Infect*".mp OR "Prosthesis Infect*".mp OR "Prosthetic elbow Infect*".mp OR "Prosthetic hip Infect*".mp OR "Prosthetic Infect*".mp OR "Prosthetic joint Infect*".mp OR "Prosthetic knee Infect*".mp OR "Prosthetic shoulder Infect*".mp))

Academic Search Premier

((TI("perioperative contamination" OR "perioperative contaminations" OR "perioperative contaminat*" OR "peri operative contamination" OR "peri operative contaminat*" OR "intraoperative contamination" OR "intraoperative contaminat*" OR "intra operative contamination" OR "intra operative contaminat*" OR "perioperative bacterial contamination" OR "perioperative bacterial contaminat*" OR "intraoperative bacterial contamination" OR "intraoperative bacterial contaminat*" OR "intra operative bacterial contamination" OR "intra operative bacterial contaminat*" OR "positive intraoperative cultures" OR "positive intraoperative culture" OR "positive intra operative cultures" OR "perioperative cultures" OR "perioperative cultures" OR "intraoperative cultures" OR "intraoperative culture" OR "intra operative cultures" OR "intra operative culture" OR "intraoperative bacterial cultures" OR "intraoperative bacterial culture" OR "intra operative bacterial cultures" OR "intraoperative bacterial cultures" OR "intraoperative bacterial culture" OR "intra operative bacterial cultures" OR "peroperative contamination" OR "peroperative contaminat*" OR "peroperative bacterial contamination" OR "peroperative bacterial contaminat*" OR "peroperative cultures" OR "peroperative cultur*" OR "per operative culture" OR "per operative cultures" OR "per operative cultur*" OR (("bacterium culture" OR "culture" OR "cultures" OR "cultured") AND ("microbial contamination" OR "contamination" OR "contaminated" OR "contaminate" OR "contaminat*" OR "sample" OR "samples") AND ("perioperative period" OR "intraoperative period" OR "intraoperative" OR "intra operative" OR "perioperative" OR "peri operative" OR "peroperative" OR "per operative" OR "during surgery" OR "during surg*" OR "during" OR "Surgical Wound Infection")) OR (("culture positive" OR "culture positiv*" OR "cultures positive" OR "cultured positive" OR "positive culture" OR "positive cultures" OR "positive cultur*") AND ("Perioperative Period" OR "Intraoperative Period" OR "intraoperative" OR "intra operative"

OR "perioperative" OR "peri operative" OR "peroperative" OR "per operative" OR "during surgery" OR "during surg*" OR "during" OR "Surgical Wound Infection")) OR "wound contamination" OR "wound contaminat*" OR "wounds contaminated" OR "wound contaminat*") OR SU("perioperative contamination" OR "perioperative contaminations" OR "perioperative contaminat*" OR "peri operative contamination" OR "peri operative contaminat*" OR "intraoperative contamination" OR "intraoperative contaminat*" OR "intra operative contamination" OR "intra operative contaminat*" OR "perioperative bacterial contamination" OR "perioperative bacterial contaminat*" OR "intraoperative bacterial contamination" OR "intraoperative bacterial contaminat*" OR "intra operative bacterial contamination" OR "intra operative bacterial contaminat*" OR "positive intraoperative cultures" OR "positive intraoperative culture" OR "positive intra operative cultures" OR "perioperative cultures" OR "perioperative cultures" OR "intraoperative cultures" OR "intraoperative culture" OR "intra operative cultures" OR "intra operative culture" OR "intraoperative bacterial cultures" OR "intraoperative bacterial culture" OR "intra operative bacterial cultures" OR "intraoperative bacterial cultures" OR "intraoperative bacterial culture" OR "intra operative bacterial cultures" OR "peroperative contamination" OR "peroperative contaminat*" OR "peroperative bacterial contamination" OR "peroperative bacterial contaminat*" OR "peroperative cultures" OR "peroperative cultur*" OR "per operative culture" OR "per operative cultures" OR "per operative cultur*" OR (("bacterium culture" OR "culture" OR "cultures" OR "cultured") AND ("microbial contamination" OR "contamination" OR "contaminated" OR "contaminate" OR "contaminat*" OR "sample" OR "samples") AND ("perioperative period" OR "intraoperative period" OR "intraoperative" OR "intra operative" OR "perioperative" OR "peri operative" OR "peroperative" OR "per operative" OR "during surgery" OR "during surg*" OR "during" OR "Surgical Wound Infection")) OR (("culture positive" OR "culture positiv*" OR "cultures positive" OR "cultured positive" OR "positive culture" OR "positive cultures" OR "positive cultur*") AND ("Perioperative Period" OR "Intraoperative Period" OR "intraoperative" OR "intra operative" OR "perioperative" OR "peri operative" OR "peroperative" OR "per operative" OR "during surgery" OR "during surg*" OR "during" OR "Surgical Wound Infection")) OR "wound contamination" OR "wound contaminat*" OR "wounds contaminated" OR "wound contaminat*") OR KW("perioperative contamination" OR "perioperative contaminations" OR "perioperative contaminat*" OR "peri operative contamination" OR "peri operative contaminat*" OR "intraoperative contamination" OR "intraoperative contaminat*" OR "intra operative contamination" OR "intra operative contaminat*" OR "perioperative bacterial contamination" OR "perioperative bacterial contaminat*" OR "intraoperative bacterial contamination" OR "intraoperative bacterial contaminat*" OR "intra operative bacterial contamination" OR "intra operative bacterial contaminat*" OR "positive intraoperative cultures" OR "positive intraoperative culture" OR "positive intra operative cultures" OR "perioperative cultures" OR "perioperative cultures" OR "intraoperative cultures" OR "intraoperative culture" OR "intra operative cultures" OR "intra operative culture" OR "intraoperative bacterial cultures" OR "intraoperative bacterial culture" OR "intra operative bacterial cultures" OR "intraoperative bacterial cultures" OR "intraoperative bacterial culture" OR "intra operative bacterial cultures" OR "peroperative contamination" OR "peroperative contaminat*" OR "peroperative bacterial contamination" OR "peroperative bacterial contaminat*" OR "peroperative cultures" OR "peroperative cultur*" OR "per operative culture" OR "per operative cultures" OR "per operative cultur*" OR (("bacterium culture" OR "culture" OR "cultures" OR "cultured") AND ("microbial contamination" OR

"contamination" OR "contaminated" OR "contaminate" OR "contaminat*" OR "sample" OR "samples") AND ("perioperative period" OR "intraoperative period" OR "intraoperative" OR "intra operative" OR "perioperative" OR "peri operative" OR "peroperative" OR "per operative" OR "during surgery" OR "during surg*" OR "during" OR "Surgical Wound Infection")) OR (("culture positive" OR "culture positiv*" OR "cultures positive" OR "cultured positive" OR "positive culture" OR "positive cultures" OR "positive cultur*") AND ("Perioperative Period" OR "Intraoperative Period" OR "intraoperative" OR "intra operative" OR "perioperative" OR "peri operative" OR "peroperative" OR "per operative" OR "during surgery" OR "during surg*" OR "during" OR "Surgical Wound Infection")) OR "wound contamination" OR "wound contaminat*" OR "wounds contaminated" OR "wound contaminat*") OR AB("perioperative contamination" OR "perioperative contaminations" OR "perioperative contaminat*" OR "peri operative contamination" OR "peri operative contaminat*" OR "intraoperative contamination" OR "intraoperative contaminat*" OR "intra operative contamination" OR "intra operative contaminat*" OR "perioperative bacterial contamination" OR "perioperative bacterial contaminat*" OR "intraoperative bacterial contamination" OR "intraoperative bacterial contaminat*" OR "intra operative bacterial contamination" OR "intra operative bacterial contaminat*" OR "positive intraoperative cultures" OR "positive intraoperative culture" OR "positive intra operative cultures" OR "perioperative cultures" OR "perioperative cultures" OR "intraoperative cultures" OR "intraoperative culture" OR "intra operative cultures" OR "intra operative culture" OR "intraoperative bacterial cultures" OR "intraoperative bacterial culture" OR "intra operative bacterial cultures" OR "intraoperative bacterial cultures" OR "intraoperative bacterial culture" OR "intra operative bacterial cultures" OR "peroperative contamination" OR "peroperative contaminat*" OR "peroperative bacterial contamination" OR "peroperative bacterial contaminat*" OR "peroperative cultures" OR "peroperative cultur*" OR "per operative culture" OR "per operative cultures" OR "per operative cultur*" OR (("bacterium culture" OR "culture" OR "cultures" OR "cultured") AND ("microbial contamination" OR "contamination" OR "contaminated" OR "contaminate" OR "contaminat*" OR "sample" OR "samples") AND ("perioperative period" OR "intraoperative period" OR "intraoperative" OR "intra operative" OR "perioperative" OR "peri operative" OR "peroperative" OR "per operative" OR "during surgery" OR "during surg*" OR "during" OR "Surgical Wound Infection")) OR (("culture positive" OR "culture positiv*" OR "cultures positive" OR "cultured positive" OR "positive culture" OR "positive cultures" OR "positive cultur*") AND ("Perioperative Period" OR "Intraoperative Period" OR "intraoperative" OR "intra operative" OR "perioperative" OR "peri operative" OR "peroperative" OR "per operative" OR "during surgery" OR "during surg*" OR "during" OR "Surgical Wound Infection")) OR "wound contamination" OR "wound contaminat*" OR "wounds contaminated" OR "wound contaminat*")) AND (TI("Prosthesis infection" OR "prosthesis related infections" OR "Prosthesis Related Infection" OR "Prosthesis Infections" OR "Prosthesis Infection" OR "Prosthetic Infections" OR "Prosthetic Infection" OR "Periprosthesis Infections" OR "Periprosthetic Infections" OR "Periprosthetic Infection" OR "Prosthetic joint Infections" OR "Prosthetic joint Infection" OR "Periprosthetic joint Infections" OR "Periprosthetic joint Infection" OR "Prosthetic knee Infections" OR "Prosthetic knee Infection" OR "Periprosthetic knee Infections" OR "Periprosthetic knee Infection" OR "Prosthetic hip Infections" OR "Prosthetic hip Infection" OR "Periprosthetic hip Infections" OR "Periprosthetic hip Infection" OR "Prosthetic shoulder Infections" OR "Prosthetic shoulder Infection" OR "Periprosthetic shoulder Infections" OR "Periprosthetic shoulder Infection" OR "Prosthetic elbow Infection"

OR "Periprosthetic elbow Infection" OR "Periprosthesis Infect*" OR "Periprosthetic elbow Infect*" OR "Periprosthetic hip Infect*" OR "Periprosthetic Infect*" OR "Periprosthetic joint Infect*" OR "Periprosthetic knee Infect*" OR "Periprosthetic shoulder Infect*" OR "Prosthesis Infect*" OR "Prosthetic elbow Infect*" OR "Prosthetic hip Infect*" OR "Prosthetic Infect*" OR "Prosthetic joint Infect*" OR "Prosthetic knee Infect*" OR "Prosthetic shoulder Infect*") OR SU("Prosthesis infection" OR "prosthesis related infections" OR "Prosthesis Related Infection" OR "Prosthesis Infections" OR "Prosthesis Infection" OR "Prosthetic Infections" OR "Prosthetic Infection" OR "Periprosthesis Infections" OR "Periprosthetic Infections" OR "Periprosthetic Infection" OR "Prosthetic joint Infections" OR "Prosthetic joint Infection" OR "Periprosthetic joint Infections" OR "Periprosthetic joint Infection" OR "Prosthetic knee Infections" OR "Prosthetic knee Infection" OR "Periprosthetic knee Infections" OR "Periprosthetic knee Infection" OR "Prosthetic hip Infections" OR "Prosthetic hip Infection" OR "Periprosthetic hip Infections" OR "Periprosthetic hip Infection" OR "Prosthetic shoulder Infections" OR "Prosthetic shoulder Infection" OR "Periprosthetic shoulder Infections" OR "Periprosthetic shoulder Infection" OR "Prosthetic elbow Infection" OR "Periprosthetic elbow Infection" OR "Periprosthesis Infect*" OR "Periprosthetic elbow Infect*" OR "Periprosthetic hip Infect*" OR "Periprosthetic Infect*" OR "Periprosthetic joint Infect*" OR "Periprosthetic knee Infect*" OR "Periprosthetic shoulder Infect*" OR "Prosthesis Infect*" OR "Prosthetic elbow Infect*" OR "Prosthetic hip Infect*" OR "Prosthetic Infect*" OR "Prosthetic joint Infect*" OR "Prosthetic knee Infect*" OR "Prosthetic shoulder Infect*") OR KW("Prosthesis infection" OR "prosthesis related infections" OR "Prosthesis Related Infection" OR "Prosthesis Infections" OR "Prosthesis Infection" OR "Prosthetic Infections" OR "Prosthetic Infection" OR "Periprosthesis Infections" OR "Periprosthetic Infections" OR "Periprosthetic Infection" OR "Prosthetic joint Infections" OR "Prosthetic joint Infection" OR "Periprosthetic joint Infections" OR "Periprosthetic joint Infection" OR "Prosthetic knee Infections" OR "Prosthetic knee Infection" OR "Periprosthetic knee Infections" OR "Periprosthetic knee Infection" OR "Prosthetic hip Infections" OR "Prosthetic hip Infection" OR "Periprosthetic hip Infections" OR "Periprosthetic hip Infection" OR "Prosthetic shoulder Infections" OR "Prosthetic shoulder Infection" OR "Periprosthetic shoulder Infections" OR "Periprosthetic shoulder Infection" OR "Prosthetic elbow Infection" OR "Periprosthetic elbow Infection" OR "Periprosthesis Infect*" OR "Periprosthetic elbow Infect*" OR "Periprosthetic hip Infect*" OR "Periprosthetic Infect*" OR "Periprosthetic joint Infect*" OR "Periprosthetic knee Infect*" OR "Periprosthetic shoulder Infect*" OR "Prosthesis Infect*" OR "Prosthetic elbow Infect*" OR "Prosthetic hip Infect*" OR "Prosthetic Infect*" OR "Prosthetic joint Infect*" OR "Prosthetic knee Infect*" OR "Prosthetic shoulder Infect*") OR AB("Prosthesis infection" OR "prosthesis related infections" OR "Prosthesis Related Infection" OR "Prosthesis Infections" OR "Prosthesis Infection" OR "Prosthetic Infections" OR "Prosthetic Infection" OR "Periprosthesis Infections" OR "Periprosthetic Infections" OR "Periprosthetic Infection" OR "Prosthetic joint Infections" OR "Prosthetic joint Infection" OR "Periprosthetic joint Infections" OR "Periprosthetic joint Infection" OR "Prosthetic knee Infections" OR "Prosthetic knee Infection" OR "Periprosthetic knee Infections" OR "Periprosthetic knee Infection" OR "Prosthetic hip Infections" OR "Prosthetic hip Infection" OR "Periprosthetic hip Infections" OR "Periprosthetic hip Infection" OR "Prosthetic shoulder Infections" OR "Prosthetic shoulder Infection" OR "Periprosthetic shoulder Infections" OR "Periprosthetic shoulder Infection" OR "Prosthetic elbow Infection" OR "Periprosthetic elbow Infection" OR "Periprosthesis Infect*" OR "Periprosthetic elbow Infect*" OR "Periprosthetic hip Infect*" OR "Periprosthetic Infect*" OR "Periprosthetic joint

Infect*" OR "Periprosthetic knee Infect*" OR "Periprosthetic shoulder Infect*" OR "Prosthesis Infect*" OR "Prosthetic elbow Infect*" OR "Prosthetic hip Infect*" OR "Prosthetic Infect*" OR "Prosthetic joint Infect*" OR "Prosthetic knee Infect*" OR "Prosthetic shoulder Infect*")))
